# The first high-quality chromosome-level genome of the Sipuncula *Sipunculus nudus* using HiFi and Hi-C data

**DOI:** 10.1038/s41597-023-02235-7

**Published:** 2023-05-25

**Authors:** Zhe Zheng, Zhuoxin Lai, Bin Wu, Xinlin Song, Wei Zhao, Ruzhuo Zhong, Jiawei Zhang, Yongshan Liao, Chuangye Yang, Yuewen Deng, Junpu Mei, Zhen Yue, Jianbo Jian, Qingheng Wang

**Affiliations:** 1 Fisheries College, Guangdong Ocean University, Zhanjiang, 524088 Guangdong China; 2 Guangdong Provincial Key Laboratory of Aquatic Animal Disease Control and Healthy Culture, Zhanjiang, 524088 Guangdong China; 3 BGI-Shenzhen, Shenzhen, 518083 Guangdong China; 4 BGI-Sanya, BGI-Shenzhen, Sanya, 572025 Hai nan China

**Keywords:** Genome, Taxonomy

## Abstract

Sipuncula is a class of exocoelomic unsegmented animals whose evolutionary relationships are unresolved. The peanut worm *Sipunculus nudus* is a globally distributed, economically important species belonging to the class Sipuncula. Herein, we present the first high-quality chromosome-level assembly of *S. nudus* based on HiFi reads and high-resolution chromosome conformation capture (Hi-C) data. The assembled genome was 1,427 Mb, with a contig N50 length of 29.46 Mb and scaffold N50 length of 80.87 Mb. Approximately 97.91% of the genome sequence was anchored to 17 chromosomes. A BUSCO assessment showed that 97.7% of the expectedly conserved genes were present in the genome assembly. The genome was composed of 47.91% repetitive sequences, and 28,749 protein-coding genes were predicted. A phylogenetic tree demonstrated that Sipuncula belongs to Annelida and diverged from the common ancestor of Polychaeta. The high-quality chromosome-level genome of *S. nudus* will serve as a valuable reference for studies of the genetic diversity and evolution of Lophotrochozoa.

## Background & Summary

Sipuncula (peanut worms) are unsegmented coelomate worms with bilaterally symmetrical bodies that are separated into a trunk and are retractable introverts^1^. Belonging to Lophotrochozoa, they are believed to form a small phylum with approximately 150 described species^2^. However, they are widely distributed in the world’s oceans at all depths, occupying most marine habitats, from intertidal zones to abyssal depths and polar to equatorial seas, including extreme environments. Over the past 520 million years, the typical features of extant Sipuncula have undergone only minor changes^3^. Therefore, Sipuncula is an exciting resource to study environmental adaptation and evolution and as an indicator of global climate change. In coastal environments, these species are critical in bioturbation to reshape the physicochemical properties and biological characteristics of the sediment^4^. In marine wetlands and pond aquaculture systems, Sipuncula and other taxa increase organic matter transport and improve ecosystem services^5^. However, gene and genome data for Sipuncula that are available in the PDB, a public database, are insufficient.

Despite the early recognition of the group, phylogenetic relationships between Sipuncula and other taxa are unclear. *Sipunculus nudus* was first described by Linnaeus in 1767 and was later considered to be a derived group of annelids^6–8^. Morphological and developmental characteristics suggest that Sipuncula is the sister group of Mollusca^9^. However, phylogenetic analyses based on mitochondrial DNA sequences as well as traits related to nervous and muscle system development indicate that Sipuncula is more closely related to Annelida than to Mollusca^10,11^. Torsten *et al*. performed phylogenomic analyses using 47,953 amino acid positions to explore the relationships among 34 annelid taxa and found that Sipuncula belongs to Annelida^12^. Therefore, the assignment of Sipuncula to annelids is still a controversial issue. Furthermore, the lack of segments in Sipuncula, which is different from other annelid taxa, provides a basis for understanding the mechanism underlying segment development. Genome sequence information is important for phylogenetic analyses. However, sequencing data for molluscs and annelids are limited. In Sipuncula, only one draft genome of *Phascolosoma esculenta* was published by Zhong *et al*.^13^. The genome data suggested that Sipuncula belonged to Annelida; however, the evolutionary relationships among Polychaeta, Oligochaeta, and Hirudinea in their reconstructed phylogenetic tree were inconsistent with previous results, making evolutionary inferences difficult. Therefore, additional genome data for Sipuncula, especially chromosome-level genome data, are needed to clarify the evolutionary relationships of lophotrochozoans and to provide genomic resources for “evo-devo” studies of body segmentation.

**Fig. 1:**
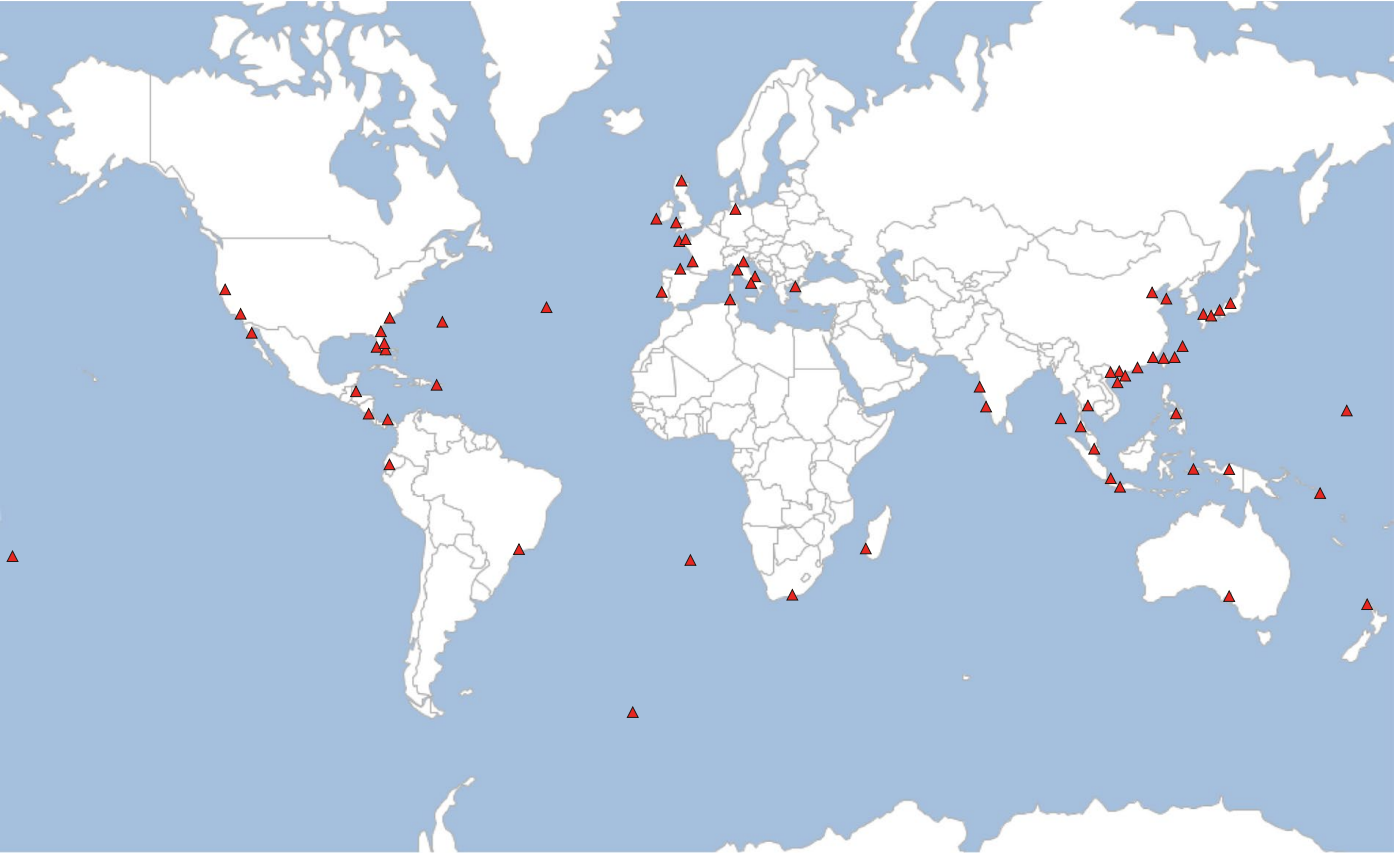
Distribution of *S. nudus* worldwide. Red triangles represent collection locations reported in the literature.

*S. nudus* is a cosmopolitan Sipuncula species that is distributed in temperate, subtropical, and tropical waters in all oceans (Fig. 1). In this study, we assembled the first high-quality genome of *S. nudus* using PacBio HiFi sequencing and high-throughput chromosome conformation capture (Hi-C). We used HiFi reads for assembly and Hi-C technology for chromosome anchoring. We obtained a contig N50 of 29.47 Mb and a scaffold N50 of 80.87 Mb for the final genome assembly, which is approximately 1,427 Mb. Using Hi-C data, 97.91% of the assembled bases were associated with the 17 chromosomes. These high-quality genomic data are expected to improve the resolution of phylogenetic analyses of Sipuncula and to provide a reference for detailed analyses of their characteristics, adaptation to complex habitats, and ecological niches.

## Methods

### Sample collection and DNA extraction

Male 2-year-old *S. nudus* samples were collected from the field of Suixi, Zhanjiang Guangdong Province, China (21°35′N, 109°81′E), and were used for whole-genome sequencing. The body wall tissue was stored in liquid nitrogen, and total genomic DNA was isolated by using the QIAGEN DNeasy Blood & Tissue Kit (QIAGEN, Shanghai, China) following the manufacturer’s instructions.

### Library construction and sequencing

Three SMRTbell libraries of circular consensus sequencing (CCS) were constructed according to the standard PacBio protocol using 15–20 kb preparation solutions (Pacific Biosciences, Menlo Park, CA, USA). Five cells were sequenced on the PacBio Sequel II platform by the CCS model (Pacific Biosciences) to generate HiFi (high-fidelity) reads. The reads were produced by calling consensus from subreads that were generated by multiple passes of the enzyme around a circularized template. This resulted in a HiFi read that was both long and accurate. In total, 103.13 Gb of HiFi reads with 72.63× coverage was generated, and the N50 value was 14,008 bp (Table 1).

**Table 1:** HiFi sequencing data statistics.

Library-ID	Raw_reads	Raw_base(Gb)	CCS_reads	CCS_base (Gb)	Clean data N50
r64048_20210717_082000-1_E01	21,040,467	253.71	1,180,145	16.16	13,696
r64048_20210720_021450-1_F01	27,767,524	348.76	1,520,742	21.02	13,819
r64048_20210723_061947-2_H01	26,285,494	321.55	1,542,752	21.21	13,745
r64048_20210802_023551-1_F01	19,891,903	283.75	1,198,971	19.33	16,123
r64048_20210806_012610-1_F01	33,585,210	392.58	2,007,850	25.41	12,656
Sum	128,570,598	1,600.35	7,450,460	103.13	14,008

Hi-C libraries were prepared as previously reported^14^. The body wall tissue cells were fixed by using formaldehyde to keep the 3D structure of DNA intact. Cells were digested with the HindIII restriction endonuclease. Biotin-labelled bases were used for end repair. The DNA fragments maintaining interaction relationships were captured to construct the Hi-C library. Finally, 289.30 Gb of high-quality Hi-C data (Q20 > 98% and Q30 > 94%) was obtained with the BGISEQ-500 sequencing platform (Table 2).

**Table 2:** Hi-C sequencing data statistics.

Library	Total Bases	%Q20	%Q30
CWHPE21060189-65	16.65	98.17	94.82
CWHPE21060189-66	18.99	98.08	94.58
CWHPE21060189-67	17.16	98.08	94.6
CWHPE21060189-68	9.41	98.03	94.44
CWHPE21060189-69	22.2	98.12	94.7
CWHPE21060189-70	16.54	98.21	94.95
CWHPE21060189-71	19.18	98.08	94.59
CWHPE21060189-72	21.44	98.13	94.71
CWHPE21060189-65	17.4	98.42	95.45
CWHPE21060189-66	19.8	98.35	95.25
CWHPE21060189-67	17.91	98.35	95.27
CWHPE21060189-68	9.87	98.31	95.13
CWHPE21060189-69	23.02	98.37	95.32
CWHPE21060189-70	17.44	98.51	95.72
CWHPE21060189-71	20.08	98.36	95.28
CWHPE21060189-72	22.24	98.39	95.39

### Genome survey and assembly

**Fig. 2:**
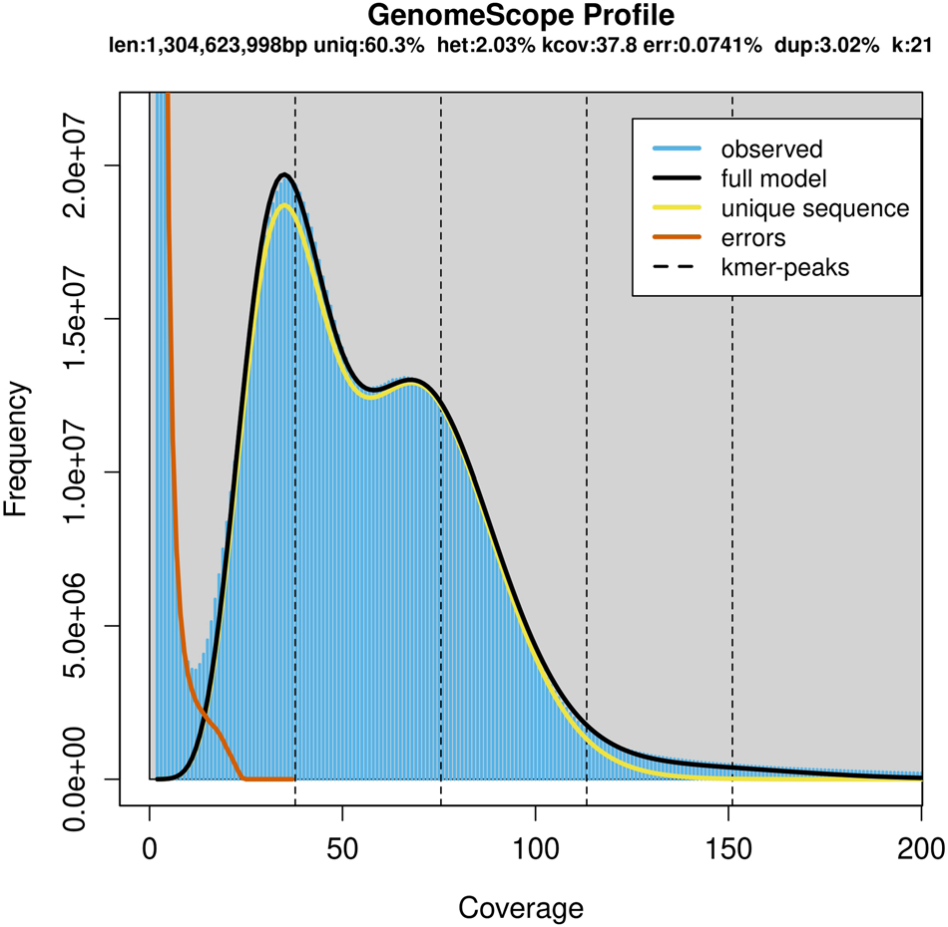
Overview of the 21-mer frequency distribution in the *S. nudus* genome. The *X*-axis is the k-mer depth, and the *Y*-axis represents the k-mer frequency for a given depth.

**Fig. 3:**
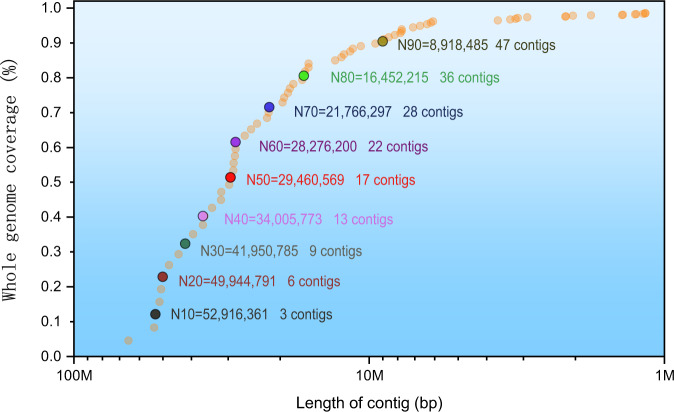
Length distribution of contigs in the preliminary genome assembly. The N50 value and number of contigs were 29,460,569 bp and 17, respectively.

The size, heterozygosity, and repeat rate of the *S. nudus* genome were estimated using the k-mer frequency method. Jellyfish^15^ and GenomeScope v.1.0^16^ were employed to calculate the K-mer frequency (k = 21), which was based on HiFi reads, and the genome size was estimated to be 1305 Mb with a peak K-mer frequency of 66X. The heterozygosity and repeat rate were 2.03% and 39.68%, respectively (Fig. 2). We first assembled the genome using HiFi reads via HiFi-asm (v0.15.1)^17^ with default parameters. After preliminary assembly, we used purge_haplotigs^18^ to purge haplotigs. The haploid genome size was 1426.68 Mb, and the N50 length was 29.46 Mb (Fig. 3 and Table 3).

**Table 3:** Genome assembly statistics using PacBio HiFi reads and Hi-C data.

	Scaffold Length(bp)	Scaffold Number	Contig Length(bp)	Contig Number
N10	98,497,835	2	52,916,361	3
N20	97,953,984	3	49,944,791	6
N30	93,061,255	5	41,950,785	9
N40	88,873,922	6	34,005,773	13
N50	80,869,746	8	29,460,569	17
N60	79,641,139	10	28,276,200	22
N70	78,550,287	12	21,766,297	28
N80	68,268,086	14	16,452,215	36
N90	63,522,239	16	8,918,485	47
Total size	1,426,776,655		1,426,684,155	
Anchored rate (%)	97.91%			

**Fig. 4:**
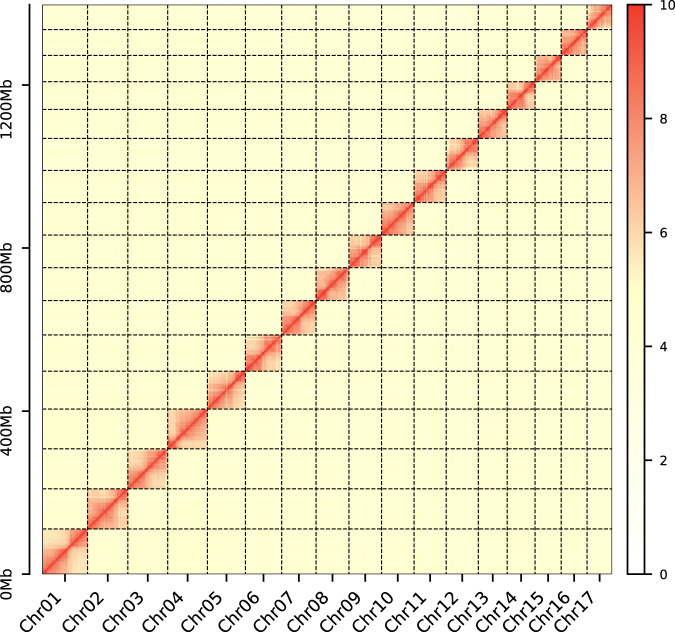
Hi-C interaction heatmap. Chr01–Chr17 indicate the 17 chromosomes. The abscissa and ordinate represent the order of each bin on the corresponding chromosome group. The colour block demonstrates the intensity of the interaction from yellow (low) to red (high).

**Fig. 5:**
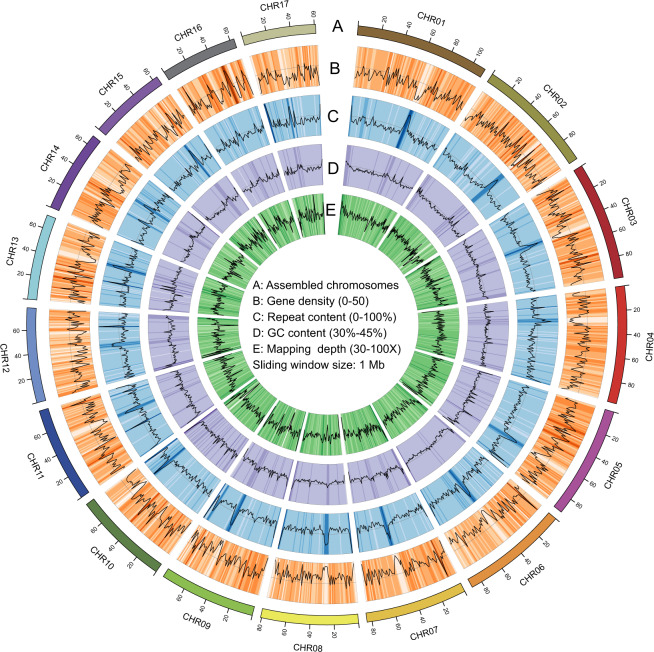
Genomic landscape of the 17 assembled chromosomes of *S. nudus*. Sliding window: 1 Mb; A: Assembled chromosomes; B: Gene density (0–50); C: Repeat content (0–100%); D: GC content (30–45%); E: Mapping depth (30–100×).

**Fig. 6:**
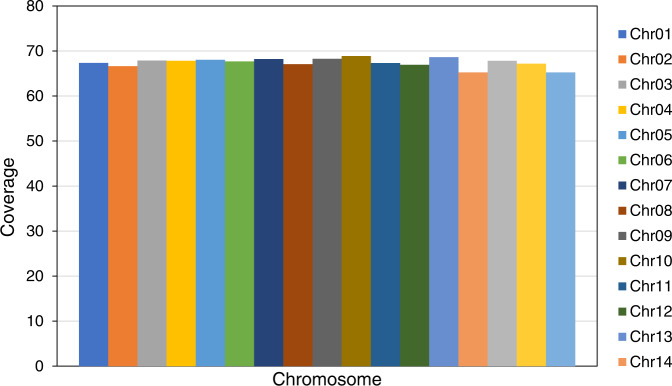
The read depth in each chromosome.

**Fig. 7:**
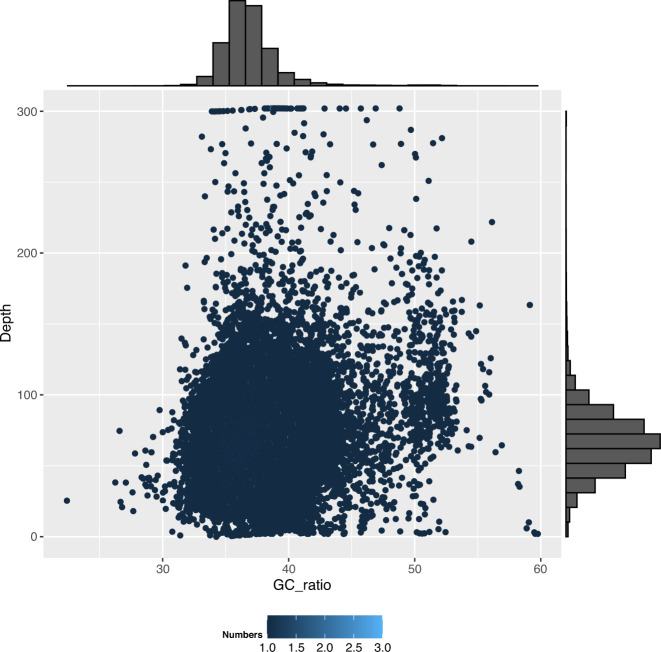
GC Content and Sequencing Depth. The x-axis represents the GC content; the y-axis represents the average depth.

**Fig. 8:**
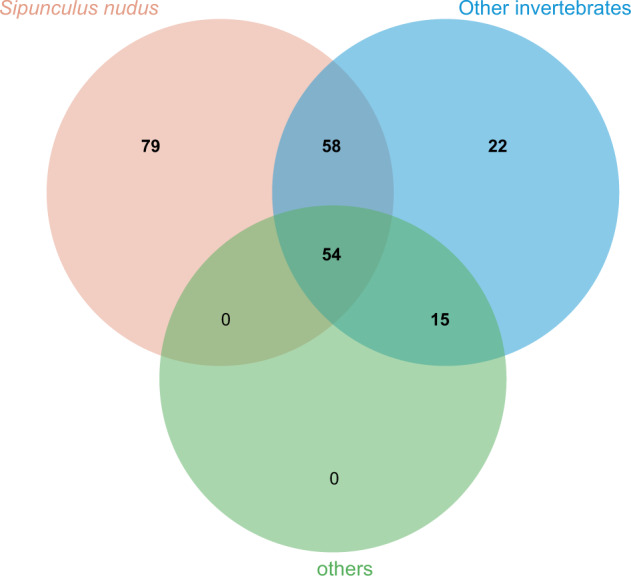
The categories of reference species in the alignments from the NT database.

The contigs were anchored to chromosomes using Hi-C data. Juicer (version 1.6)^19^ was used to align the double-ended sequencing data against the assembled genome to complete the evaluation of the Hi-C library. The 3D-DNA pipeline^20^ under default parameters without breaking contigs was chosen to generate the final chromosome-level scaffolds. Manual checking and refinement of the draft assembly were carried out via Juicebox Assembly Tools (https://github.com/aidenlab/Juicebox, v1.1108). A heatmap of the Hi-C assembly interaction bins indicated that the quality of the genome assembly was excellent (Fig. 4). The length of the final assembled genome was 1,426,776,655 bp, with a contig N50 of 29,460,569 bp and scaffold N50 of 80,869,746 bp (Table 3 and Fig. 3). Approximately 1,397 Mb (97.91%) of the contig sequences were anchored to 17 chromosomes (Table 4), which is consistent with the known karyotype in our previously published manuscript^21^. Using the minimap2 (v2.17, parameters: -a -x map-pb)^22^ alignment results and the HiFi data, we used BamDeal (https://github.com/BGI-shenzhen/BamDeal) to evaluate the mapping rate and coverage and obtained estimates of 99.95% and 99.73%, respectively. The CIRCOS tool^23^ was used to visualize the 17 chromosomes, GC content, read depth and mapping depth (Fig. 5). The average depth of each chromosome was calculated and is shown in Fig. 6. Seventeen chromosomes had a comparable sequencing depth, and there was no whole chromosome with half the read depth. Therefore, XY- or ZW-type sex chromosomes did not exist in the assembled chromosomes of *S. nudus*. Based on 20-kb nonoverlapping sliding windows in the chromosomes to calculate the GC content and read average depth, there was a small cluster of sliding windows (a total of 11.6 Mb with 581 sequences) that exhibited relatively high GC contents ( > 48%) but with a normal sequencing depth (Fig. 7). By extracting those block sequences with high GC contents and mapping them to the NT database (Nucleotide Sequence Database) using MegaBlast (parameter: −e 1e-5), the alignments with identity >90% and coverage length >100 bp were filtered. The matched reference species in the alignments from the NT database were grouped into three categories: the *S. nudus* species, the species of other invertebrates, and all other species except the two mentioned above. All the matched sequences (228) could be correlated with *S. nudus* or other invertebrate species (Fig. 8), which demonstrated that the sequence blocks with high GC content and normal depth in chromosomes were from the *S. nudus* species rather than from contamination or cobionts.

**Table 4:** Genome chromosome length statistics.

Chromosome ID	Length (bp) without N
Chr01	110,913,107
Chr02	98,497,835
Chr03	97,953,984
Chr04	97,527,750
Chr05	93,061,255
Chr06	88,873,922
Chr07	84,190,596
Chr08	80,869,746
Chr09	79,822,965
Chr10	79,641,139
Chr11	79,021,090
Chr12	78,550,287
Chr13	70,941,565
Chr14	68,268,086
Chr15	64,178,801
Chr16	63,522,239
Chr17	61,128,531
Total anchored length	1,396,962,898

### Repeat annotation

Prior to gene prediction, we identified the repetitive elements in the genome of *S. nudus* by using a combination of homology-based and ab initio-based methods. To identify tandem repeats, we used Tandem Repeats Finder v4.09^24^. For the homology-based method, transposable elements were identified by RepeatMasker v4.0.7 (-nolow -no_is -norna -engine ncbi -parallel 1) and RepeatProteinMask v4.0.7 (-engine ncbi -noLowSimple -pvalue 0.0001)^25^ against the TE protein databases and RepBase library v21.12^26^. For the ab initio-based method, LTR_FINDER v1.06^27^ and RepeatModeler v1.0.8 (http://repeatmasker.org/RepeatModeler/) with default parameters were used to build the *de novo* library before RepeatMasker v4.0.7 was used to classify the different categories of repetitive elements against this library. The final repetitive elements were identified by integrating the results of these methods according to sequence overlap, revealing that nearly half of the genome consists of repetitive elements (Tables 5, 6; Fig. 5).

**Table 5:** Genome repetitive element statistics.

Type	Repeat Size(bp)	% of genome
TRF	188,922,055	13.24
RepeatMasker	73,550,643	5.16
RepeatProteinMask	36,744,549	2.58
De novo	587,069,040	41.15
Total	683,551,259	47.91

**Table 6:** TE type statistics.

Type		Length(bp)	% of genome
Retro	Retro/LTR/Copia	386414	0.31
Retro	Retro/LTR/Gypsy	98040062	6.87
Retro	Retro/LTR/Other	126593667	8.87
Retro	Retro/SINE	11794880	0.83
Retro	Retro/LINE	80839275	5.67
Retro	Retro/Other	0	0
DNA	DNA/EnSpm	18750800	1.31
DNA	DNA/Harbinger	4364285	0.31
DNA	DNA/hAT	25218268	1.77
DNA	DNA/Helitron	240087518	16.83
DNA	DNA/Mariner	704157	0.05
DNA	DNA/MuDR	4935134	0.35
DNA	DNA/P	3555129	0.25
DNA	DNA/Other	86203017	6.04
Other	—	4598687	0.32
Unknown	—	28683040	2.01

### Gene prediction

Gene annotation was performed by integrating homology-, *de novo*- and transcriptome-based information. We used the annotation data from three closely related species (*Caenorhabditis elegans*, *Capitella teleta*, and *Helobdella robusta*) for homology prediction. The MAKER tool^28^ was used to integrate the annotation data from the three related species and the transcriptome data from *S. nudus*. Based on AED values from MAKER, 2000 genes with complete structures were selected and used to train the *de novo* prediction tools Augustus^29^ and Snap^30^ to construct *de novo* models. Finally, all data were integrated using MAKER^28^. The final comprehensive gene set contained 28,749 genes (Table 7).

**Table 7:** General statistics of predicted protein-coding genes.

	Gene set	Number	Average				
			gene length (bp)	CDS length (bp)	exon per gene	exon length (bp)	intron length (bp)
De novo	Augustus	68,080	8,282.32	1,259.15	4.86	258.87	1,817.59
De novo	Snap	181,461	9,239.83	745.85	4.76	156.63	2,257.89
Homolog	*C. elegans*	23,278	1,875.48	458.17	1.58	238.28	2,448.04
Homolog	*C. teleta*	195,605	1,581.97	447.14	1.38	324.3	2,996.03
homolog	*H. robusta*	154,743	996.31	328.81	1.25	262.62	2,648.64
RNA-seq	Transcript	706,396	5,518.82	1,231.32	2.4	513.49	3,067.00
Final	—	28,749	13,739.77	1,383.54	6.84	202.36	2,116.89

Note: Three approaches were employed for gene prediction: Homologue (*C. elegans*, *C. teleta*, and *H. robusta*), *de novo* (AUGUSTUS and Snap) and RNA-seq transcripts. The results can be consolidated using the program MAKER.

### Gene function annotation

Gene function annotation was performed based on sequence similarity and domain conservation. First, the protein-coding genes of S. *nudus* were aligned against the KEGG^31^, SwissProt^32^, TrEMBL^33^, GO^34^, KOG (ftp://ftp.ncbi.nih.gov/pub/COG/KOG/), and Nr (https://ftp.ncbi.nlm.nih.gov/blast/db/FASTA/nr.gz) databases by using BLASTP with an E-value threshold of 1e-5. Subsequently, the best match from the alignment was used to predict gene functions. Second, searches performed using InterProScan (51.0–55.0)^35^ against the following databases were used to identify the motif and domain: PANTHER^36^, Pfam^37^, PRINTS^38^, ProDom^39^, SUPERFAMILY^40^, and SMART^41^. In total, 88.75% of the predicted genes were functionally annotated (Table 8).

**Table 8:** Functional annotation statistics.

Values	Total	Nr	SwissProt	KEGG	KOG	TrEMBL	Interpro	GO	Overall
Number	28,749	24,425	19,425	20,229	18,150	24,403	24,027	16,104	25,514
Percentage (%)	—	84.96	67.57	70.36	63.13	84.88	83.58	56.02	88.75

Note: Seven protein databases were used to predict gene functions: Nr, InterPro, Gene Ontology, KOG, KEGG, SwissProt and TrEMBL. The table shows the numbers of genes that were matched to each database.

## Data Records

The National Center for Biotechnology Information (NCBI) BioProject accession number for the sequence reported in this paper is PRJNA901211. The raw data for Hi-Fi and Hi-C sequencing were submitted to NCBI SRA (accession number SRP408321; https://identifiers.org/ncbi/insdc.sra:SRP408321)^42^ and deposited in the CNGB Sequence Archive (CNSA) of the China National GeneBank DataBase (CNGBdb) (accession number CNR0640303-CNR0640323; https://db.cngb.org/search/project/CNP0003624/)^43^. The assembled genome sequence was deposited into NCBI under accession number JAPPUL000000000^44^. The assembled genome, gene structure annotation, repeat predictions, gene function annotation, KEGG analysis of expanded genes and positively selected gene data were deposited in the China National GeneBank DataBase (CNGBdb) under the project with accession number CNP0003624.

## Technical Validation

### Genome assembly and gene prediction quality assessment

The BUSCO pipeline was used to evaluate the completeness of the genome assembly and gene set based on a benchmark of 255 conserved genes in eukaryota_odb10 (creation date: 2020-09-10, number of genomes: 70, number of BUSCOs: 255). In total, 97.7% of the 255 expected conserved genes were identified as complete, and 2% were identified as fragmented. Furthermore, we used minimap2 (v2.17, parameters: -a -x map-pb)^22^ to align the assembly with the HiFi data, and the mapping rate and coverage rate were estimated to be 99.95% and 99.73%, respectively. The BUSCO (v5)^45^ results supported the completeness of the assembly; 97.7% of 255 conserved genes were identified as complete by using eukaryota_odb10 (Table 9). The BUSCO results and alignment results indicated high genome assembly completeness and correctness.

**Table 9:** Evaluation of genome assembly completeness.

Type	Number	Percentage
Complete BUSCOs (C)	249	97.65%
Complete and single-copy BUSCOs (S)	245	96.08%
Complete and duplicated BUSCOs (D)	4	1.57%
Fragmented BUSCOs (F)	5	1.96%
Missing BUSCOs (M)	1	0.39%
Total	255	

### Comparative genomic analysis

**Fig. 9:**
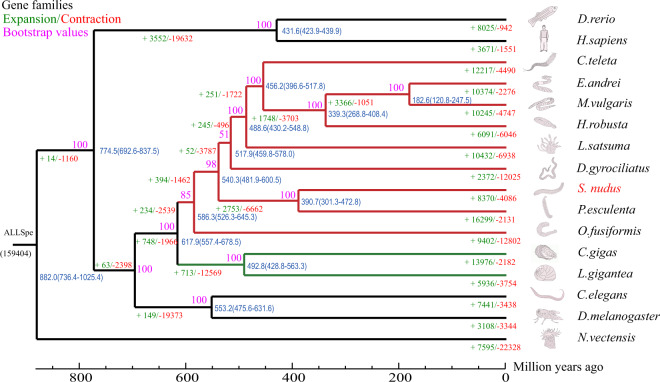
Phylogenetic tree of *S. nudus* and other species. The red branch represents Annelida, and the green branch represents Mollusca.

**Fig. 10:**
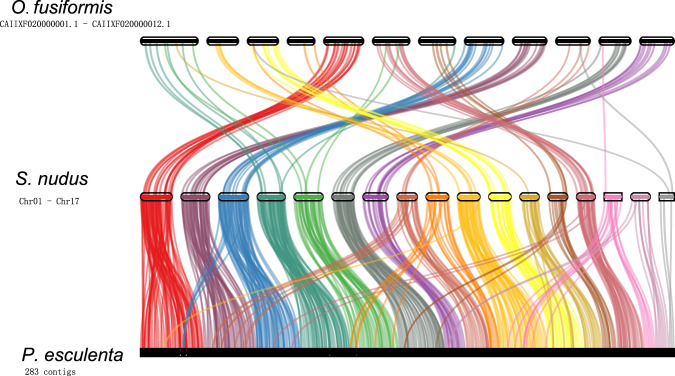
Genome synteny analysis between *S. nudus* and *P. esculenta* as well as *S. nudus* and *O. fusiformis*. Twelve chromosomes of *O. fusiformis*, seventeen chromosomes of *S. nudus* and 283 contigs of *P. esculenta* were shown.

The protein-coding genes of *S. nudus* and 15 additional species were used to identify orthologous gene groups. The reference protein sequences of the following 15 species were obtained: *Caenorhabditis elegans* (Ensembl Release 10), *Danio rerio* (Ensembl Release 10), *Homo sapiens* (Ensembl Release 10), *Drosophila melanogaster* (Ensembl Release 10), *Capitella teleta* (NCBI: GCA_000328365.1), *Crassostrea gigas* (NCBI: GCF_902806645.1), *Dimorphilus gyrociliatus* (NCBI: GCA_904063045.1), *Eisenia andrei* (ngdc.cncb.ac.cn: PRJCA002327), *Helobdella robusta* (NCBI: GCF_000326865.1), *Lamellibrachia satsuma* (NCBI: GCA_022478865.1), *Lottia gigantea* (NCBI: GCF_000327385.1), *Metaphire vulgaris* (NCBI: GCA_018105865.1), *Owenia fusiformis* (NCBI: GCA_903813345.2), *Phascolosoma esculenta* (https://figshare.com/: PRJNA819496), and *Nematostella vectensis* (NCBI: GCF_932526225.1) as the outgroup. To perform the gene family analysis, orthogroups of the 16 species were identified using OrthoFinder (v2.3.11) with default parameters^46^. After analysis of the gene family, 416,469 genes from the 16 species were grouped into 30,677 gene families. The results revealed that 717 gene families that involved 4,217 genes were unique in *S. nudus*. The gene families and genome statistics of all the species are shown in Table 10. Among the orthologous genes in the 16 species, a total of 255 single-copy genes were identified. The single-copy orthologues were aligned using MUSCLE (v3.7)^47^ with default parameters, and then the aligned protein sequences were reverse translated into codon sequences. The alignments were then concatenated to generate a superalignment matrix for phylogenetic reconstruction based on the maximum-likelihood (ML) method using IQ-TREE (v1.6.12)^48^, with the best-fit evolutionary substitution model being determined using ModelFinder^49^. Divergence times for each node in the phylogenetic tree were estimated using MCMCtree, which is implemented in PAML package v4.8a^50^, under the following parameters: -nsample 100000, -rootage 800, and -burnin 500000. The calibration times were obtained from TimeTree^51^: 630.0–830.0 million years ago (Ma) for *Caenorhabditis elegans* and *Homo sapiens*, 424.2–440.0 Ma for *Danio rerio* and *Homo sapiens*, and 545.0–681.5 Ma for *Capitella teleta* and *Crassostrea gigas*. The phylogenetic tree representing the evolutionary relationships among Mollusca, Annelida and Sipuncula is shown in Fig. 9. Gene collinearity, which shows the preservation of ancestral genome structure in the modern genome, is an important means of unveiling genomic evolution. Thus, MCscan (Python version)^52^ was used for the genomic synteny analysis between *S. nudus*, *O. fusiformis* and *P. esculenta*. The collinearity figure was drawn based on the homologous blocks with ≥ 4 gene collinear pairs between species by JCVI (https://github.com/tanghaibao/jcvi) (Fig. 10). Regarding intergenomic gene collinearity, 109 blocks containing 508 collinear gene pairs were revealed between *S. nudus* and *O. fusiformis*, and 622 blocks containing 3248 collinear gene pairs were revealed between *S. nudus* and *P. esculenta*, showing similar collinearity between the two Sipuncula species.

**Table 10:** The gene family statistics.

Species	Genome size (Mb)	Number of genes	Number of genes in orthogroups	Number of unassigned genes	Number of species-specific orthogroups	Number of genes in species-specific orthogroups
*Caenorhabditis elegans*	103	20082	15505	4577	1274	7402
*Capitella teleta*	333	31978	28405	3573	1010	5483
*Crassostrea gigas*	587	31371	29446	1925	1450	8232
*Danio rerio*	1405	25444	24064	1380	427	2565
*Drosophila melanogaster*	139	13857	11266	2591	532	2365
*Eisenia andrei*	1315	31817	29136	2681	836	5059
*Helobdella robusta*	235	23426	18768	4658	453	3645
*Homo sapiens*	2866	20212	19101	1111	300	2071
*Lottia gigantea*	359	23827	19834	3993	567	3587
*Metaphire vulgaris*	728	28855	26536	2319	517	3189
*Nematostella vectensis*	269	21752	19474	2278	924	4094
***Sipunculus nudus***	**1427**	**28749**	**26510**	**2239**	**717**	**4217**
*Dimorphilus gyrociliatus*	78	14204	12747	1457	273	1274
*Lamellibrachia satsuma*	665	32394	25643	6751	911	4227
*Owenia fusiformis*	500	27032	23803	3229	1123	5656
*Phascolosoma esculenta*	1709	41469	36394	5075	1563	6461

The time-calibrated phylogenetic tree was used to assess gene family expansions and contractions using CAFÉ 4.2.1^53^ with a random birth-and-death model with lambda. In total, 543 and 97 significantly expanded and contracted gene families were identified, respectively (P < 0.05). GO and KEGG enrichment analyses of the expanded gene families revealed that these families were mainly involved in pathways that are related to apoptosis, detoxification, the immune response, amino acid and fatty acid metabolism anion, oxidative stress, and energy metabolism.

PSGs (positively selected genes) were predicted using branch-site likelihood ratio tests for single-copy gene families with a conservative 10% false discovery rate (FDR) criterion^54^. We used proteins from *S. nudus*, *C. teleta*, *E. Andrei, L. satsuma, O. fusiformis*, and *P. esculenta* to extract 3,192 one-to-one orthologous genes using the OrthoFinder (v2.3.11) pipeline. The one-to-one orthologous genes were then used to generate multiple sequence alignments by using PRANK (v. 121002)^55^. The *d*_N_/*d*_S_ ratios of the codons were calculated using the branch-site model of Codeml in the PAML package^50^, in which *S. nudus* was set as the foreground branch and the other five taxa as background branches. Using a likelihood ratio test (LRT) of ≤0.05 and an FDR of ≤0.05 as thresholds, 326 PSGs were identified in the *S. nudus* genome. These PSGs were significantly enriched in the terms “Spliceosome,” “Base excision repair,” “DNA replication,” and “Cell cycle” in the KEGG pathway enrichment analysis.

In summary, we obtained the high-quality chromosome-level genome of *S. nudus*, which contributes to our understanding of the evolutionary status of Sipuncula and the evolutionary relationship among the subgroups of the phylum Annelida. Gene family expansion and extraction and genomic synteny analyses revealed the potential adaptation mechanism of Sipuncula to different living environments.

## Usage Notes

All analyses were run on Linux systems, and the optimal parameters are given in the Code availability section.

## Data Availability

No specific code or script was used in this work. Commands used for data processing were all executed according to the manuals and protocols of the corresponding software.
